# Supplementary material for: Defective IgG Class Switching in the Spleen of TRAF5-Deficient Mice Reveals a Role for TRAF5 in CD40-Mediated B Cell Responses During Obesity-Associated Inflammation
Source: Int J Mol Sci. 2025 Sep 28;26(19):9494. doi: 10.3390/ijms26199494 (PMC12525364; doi:10.3390/ijms26199494)
Supplement: Supplementary file 1 [file ijms-26-09494-s001.zip › ijms-3818178-supplementary.pdf]

Table S1. Antibodies used in this study.

Antibody	Identifier, Source
Phycoerythrin (PE) /Cyanine7 (Cy7) conjugated anti mouse/human CD45R/B220	103221, BioLegend, San Diego, CA, USA
PE conjugated anti-mouse CD1d (CD1.1, Ly-38)	123509, BioLegend, San Diego, CA, USA
Alexa Fluor® 488 conjugated anti-mouse CD23	101609, BioLegend, San Diego, CA, USA
Fluorescein isothiocyanate (FITC) conjugated anti-mouse CD4	100510, BioLegend, San Diego, CA, USA
PE conjugated anti-mouse CD8a	100707, BioLegend, San Diego, CA, USA
Allophycocyanin (APC) conjugated anti-mouse/human CD44	103011, BioLegend, San Diego, CA, USA
PE conjugated anti-mouse CD62L	104407, BioLegend, San Diego, CA, USA
Purified-Goat anti-mouse Ig, Human ads-UNLB	1010-01, Southern Biotech, Birmingham, AL, USA
Alkaline Phosphatase (AP) conjugated Goat anti-mouse IgG2c	1078-04, Southern Biotech, Birmingham, AL, USA
AP conjugated Goat anti-mouse IgG3	1100-04, Southern Biotech, Birmingham, AL, USA
AP conjugated Goat anti-mouse IgG1	1070-04, Southern Biotech, Birmingham, AL, USA
AP conjugated Goat anti-mouse IgM	1020-04, Southern Biotech, Birmingham, AL, USA
AP conjugated Goat anti-mouse IgA	1040-04, Southern Biotech, Birmingham, AL, USA

Table S2. Primers used in this study.

Gene	**Forward primer**	**Reverse primer**
Aicda	5’-CGTGGTGAAGAGGAGAGATAGTG-3'	5’-CAGTCTGAGATGTAGCGTAGGAA-3'
Bcl6	5'-CCGGCACGCTAGTGATGTT-3'	5'-TGTCTTATGGGCTCTAAACTGCT-3'
Cd40lg	5'-ACACGTTGTAAGCGAAGCCA-3'	5'-AATGGGCGTTGACTCGAAGG-3'
Ebi3	5'-TCATTGCCACTTACAGGCTCG-3'	5'-TGATGATTCGCTCAGCCACAA-3'
Foxp3	5'-CCCATCCCCAGGAGTCTTG-3'	5'-ACCATGACTAGGGGCACTGTA-3'
Gata3	5'-GGCAGAACCGGCCCCTTATC-3'	5'-TGGTCTGACAGTTCGCGCAG-3'
Ifng	5'-GGATGCATTCATGAGTATTGC-3'	5'-CCTTTTCCGCTTCCTGAGG-3'
Il4	5'-AGATCATCGGCATTTTGAACG-3'	5'-TTTGGCACATCCATCTCCG-3'
Il6	5'-CTGGGAAATCGTGGAAATGAG-3’	5'-GGACTCTGGCTTTGTCTTTCTTG-3'
Il10	5'-TGCTCCTAGAGCTGCGGACT-3'	5'-AGGCTTGGCAACCCAAGTAACC-3'
Il17a	5'-TTTAACTCCCTTGGCGCAAAA-3'	5'-CTTTCCCTCCGCATTGACAC-3'
Il17f	5'-TGAAGTGCACCCGTGAAACA-3'	5'-CCCTCAGAATGGCAAGTCCC-3'
Il21	5'-ACTCAGTTCTGGTGGCATGG-3'	5'-GCTGATAGAAGTTCAGGATCCAAGT-3'
Il23r	5'-CTGCATGTGGTGATAGCCCTT-3'	5'-CCCACATGTCACCAGAGCAG-3'
Il27	5'-GGCCATGAGGCTGGATCTC-3'	5'-AACATTTGAATCCTGCAGCCA-3'
Rn18s	5’-CGTTCTTAGTTGGTGGAGC-3‘	5’-TAAGGGCATCACAGACCT-3'
Rorc	5'-TCGACAAGGCCTCCTAGCCA-3'	5'-CTTGGACCACGATGGGGTGG-3'
Socs3	5'-AGGCCGGAGATTTCGCTTCG-3'	5'-CGGGAAACTTGCTGTGGGTGA-3'
Stx5a	5'-GCACACATGGTTAAAGAACAGGAG-3'	5'-CAGGCAAGGAAGACCACAAAG-3'
Tbx21	5'-GGTTGGAGGTGTCTGGGAAGC-3'	5'-GCCACGGTGAAGGACAGGAAT-3'
Tnf	5'-CTGAGGTCAATCTGCCCAAGTAC-3'	5'-CTTCACAGAGCAATGACTCCAAAG-3'

Figure S1

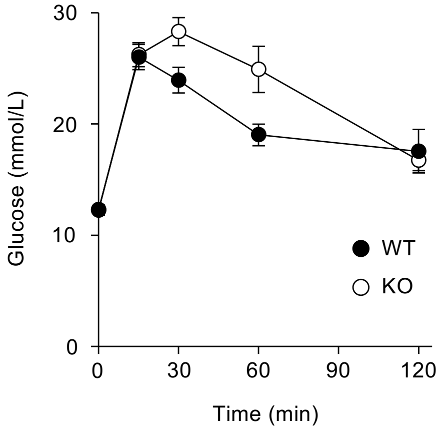

Figure S1. Glucose tolerance test in *Traf5*$^{+/+}$ (WT) and *Traf5*$^{-/-}$ (KO) mice fed a high-fat diet for 27 weeks and fasted for 4 hours. Mice were intraperitoneally injected with 2 g/kg glucose (049-31165, FUJIFILM Wako, Osaka, Japan) after the fasting period. Blood glucose levels were measured using a glucose meter (ACCU-CHEK® Guide, 000485, Roche DC Japan Corporation, Tokyo, Japan) at baseline and at 15, 30, 60, and 120 minutes after glucose administration. Data are presented as as mean ± standard error of the mean (WT: n = 6, KO: n = 8). Statistical significance was determined using the Tukey–Kramer test.

Figure S2

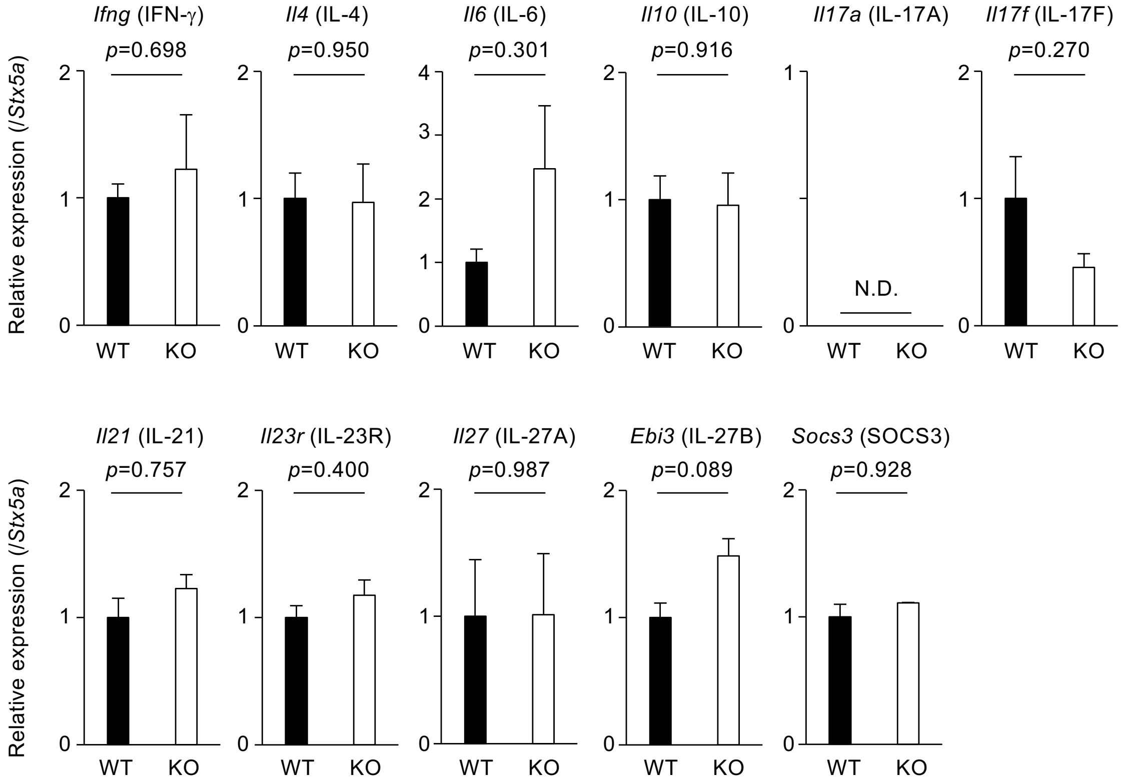

Figure S2. Expression profiles of cytokine-related genes in splenocytes from *Traf5*$^{+/+}$ (WT) and *Traf5*$^{-/-}$ (KO) mice fed a high-fat diet. The expression levels of *Ifng, Il4, Il6, Il10, Il17a, Il17f, Il21, Il23r, Il27, Ebi3,* and *Socs3* were quantified by real-time RT-PCR in splenocytes from WT and KO mice, as shown in Figures 1B and 2A. Data are normalized to *Stx5a* expression and presented as mean ± standard error of the mean (WT (n = 3), KO (n = 3)). For each gene, the expression level in WT mice was set to 1. *P*-values were calculated using Student's *t*-test. N.D., not detected.

Figure S3

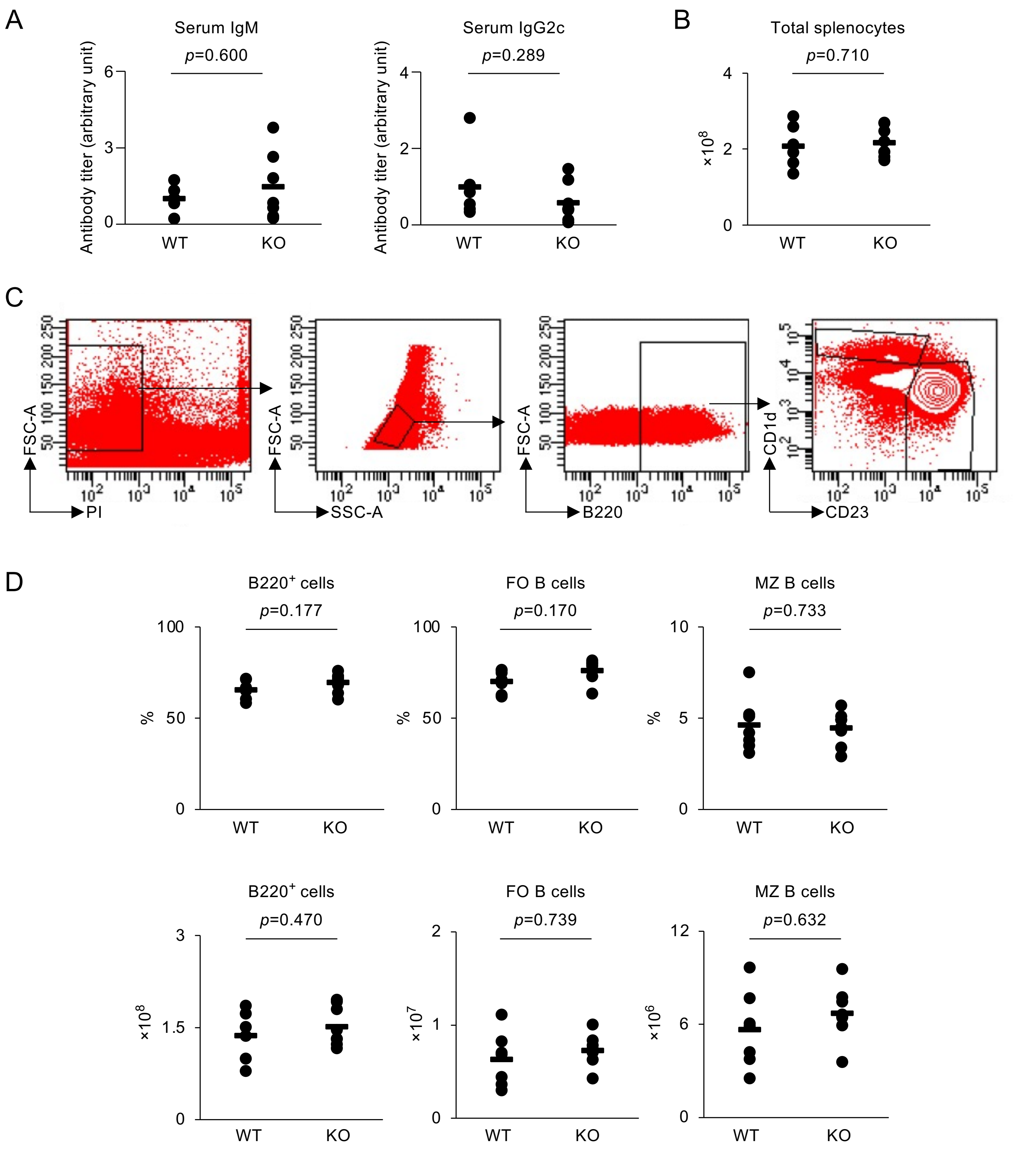

Figure S3. Profiles of serum antibodies and splenic B cell populations in *Traf5*$^{+/+}$ (WT) and *Traf5*$^{-/-}$ (KO) mice fed a high-fat diet (HFD). (**A**) Serum IgM and IgG2c titers in WT (n = 6) and KO (n = 8) mice fed an HFD, as shown in Figures 1B and 2A, were measured by ELISA. (**B**) Total splenocyte counts. (**C**) Gating strategy used to identify propidium iodide (PI)-negative live B cell populations in the spleen by flow cytometry: follicular (FO) B cells (B220^{+}CD23^{high}CD1d^{low}) and marginal zone (MZ) B cells (B220^{+}CD23^{low}CD1d^{high}). (**D**) Percentages and absolute numbers of B220^{+} cells, FO B cells, and MZ B cells in the spleen. Bars represent mean values; dots indicate individual mice (WT (n = 6), KO (n = 8)), as shown in Figures 1B and 2A. *P*-values were calculated using Student's *t*-test.

Figure S4

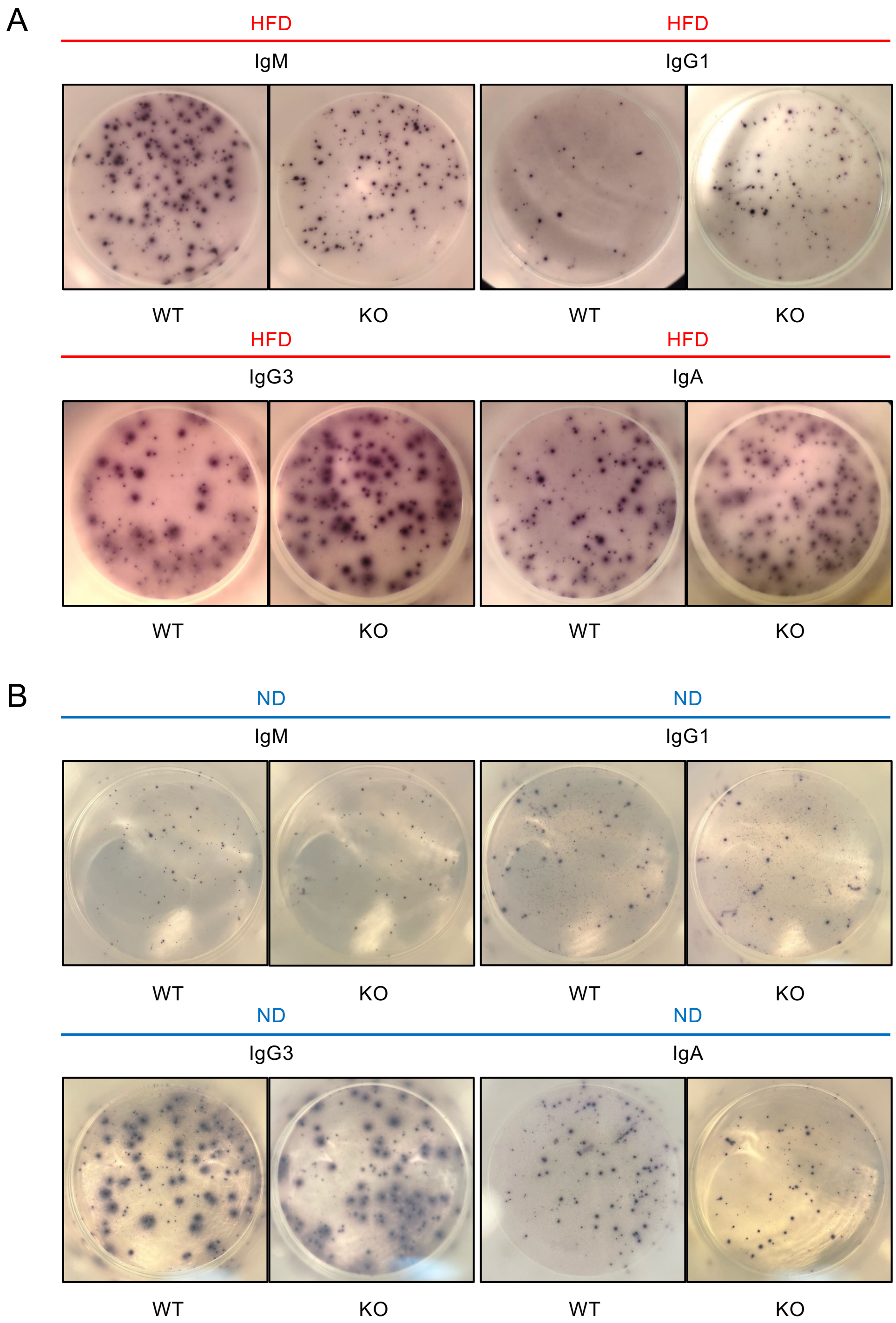

Figure S4. Representative ELISPOT images showing IgM-, IgG1-, IgG3, and IgA-producing splenocytes isolated from *Traf5*$^{+/+}$ (WT) and *Traf5*$^{-/-}$ (KO) mice. Panels show data from mice fed either (**A**) a high-fat diet (HFD) or (**B**) a normal diet (ND), corresponding to the experimental conditions described in Figure 2.

Figure S5

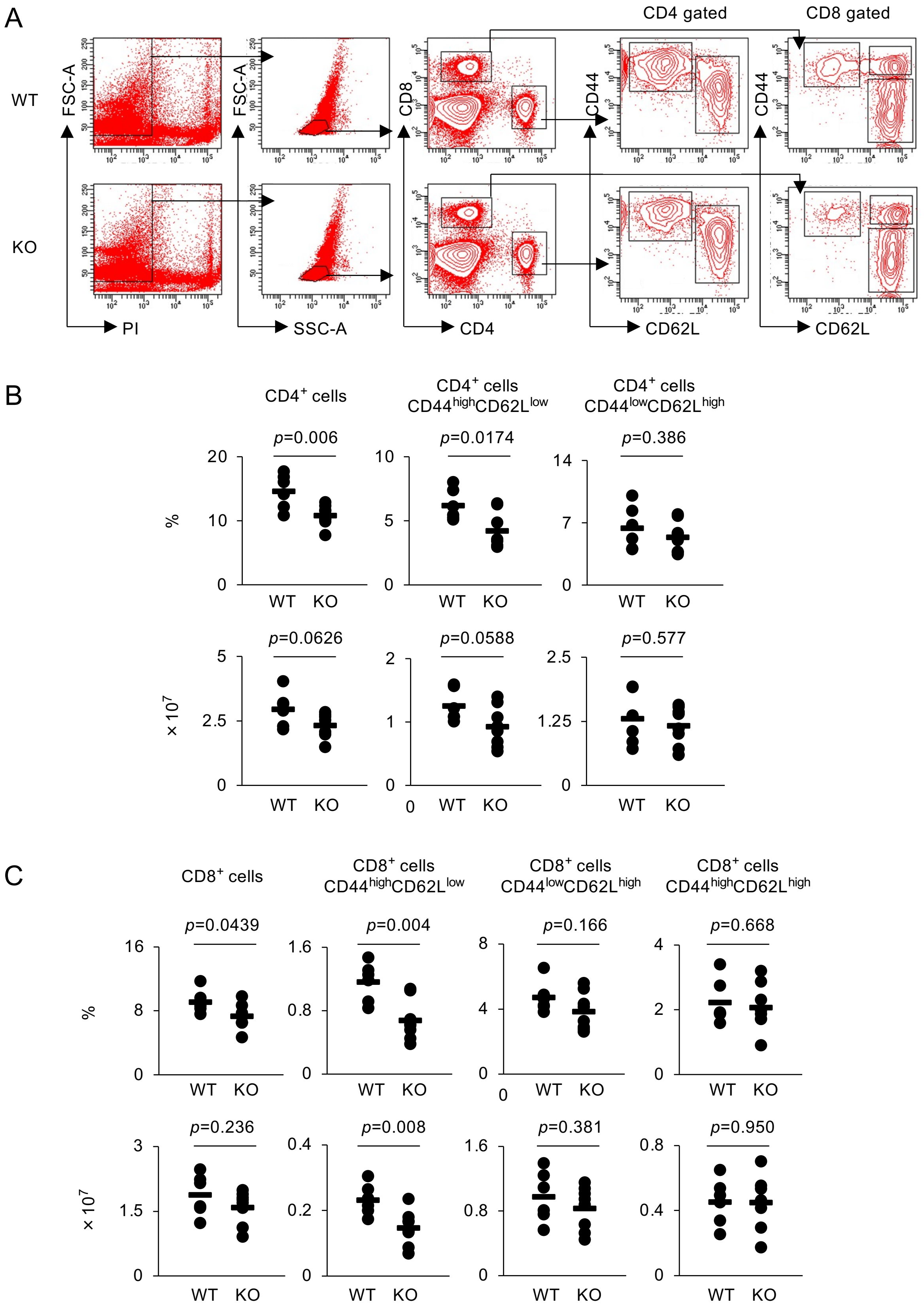

Figure S5. Profiles of splenic T cell populations in *Traf5*$^{+/+}$ (WT) and *Traf5*$^{-/-}$ (KO) mice fed a high-fat diet (HFD). (**A**) Gating strategy used to identify propidium iodide (PI)-negative live T cell populations in the spleen by flow cytometry: CD4^{+}, CD8^{+}, CD4^{+}CD44^{high}CD62L^{low} (effector memory), CD4^{+}CD44^{low}CD62L^{high} (naïve), CD8^{+}CD44^{high}CD62L^{low} (effector memory), CD8^{+}CD44^{low}CD62L^{high} (naïve), and CD8^{+}CD44^{high}CD62L^{high} (central memory) T cells. (**B**, **C**) Percentages and abusolute numbers of each CD4^{+} (B) and CD8^{+} (C) T cell subset in the spleen. Bars represent mean values; dots indicate individual mice (WT (n = 6), KO (n = 8)), as shown in Figures 1B and 2A. *P*-values were calculated using Student's *t*-test.

Figure S6

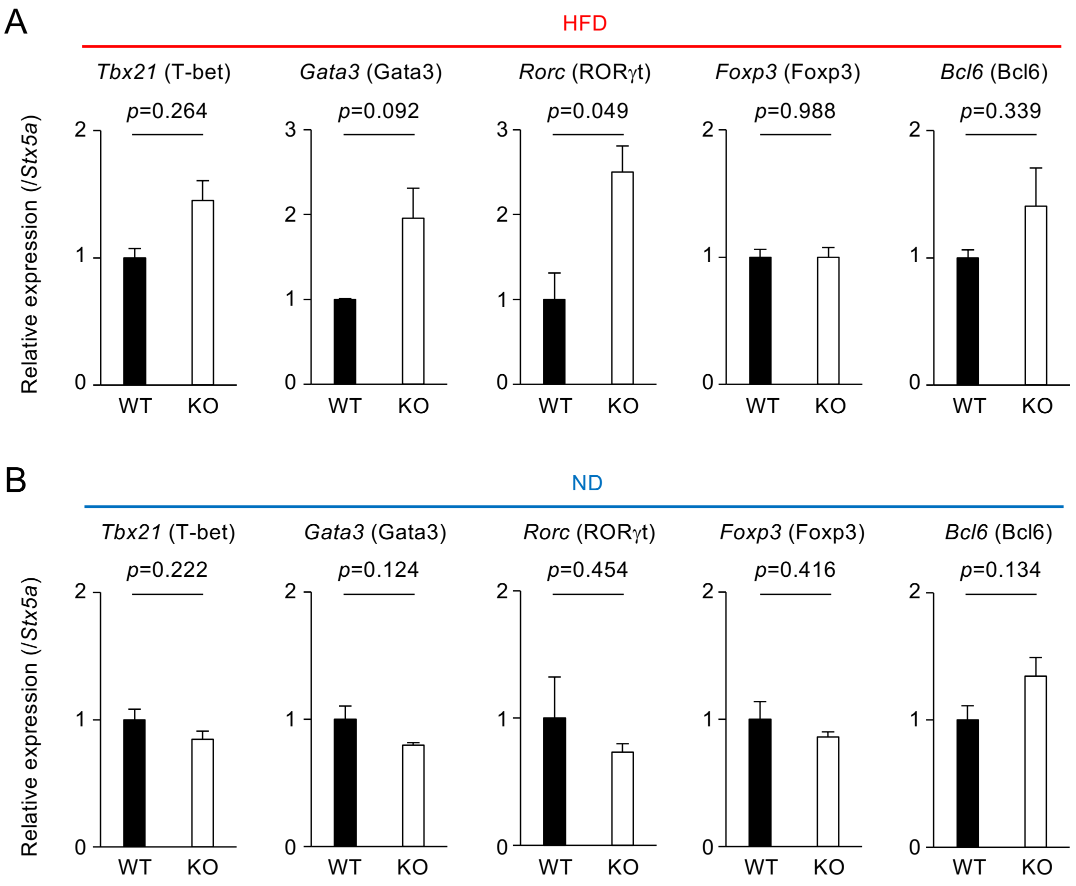

Figure S6. The Th17 lineage-specific transcription factor *Rorc* (RORγt) is significantly upregulated in the spleen of *Traf5*$^{-/-}$ (KO) mice fed a high-fat diet (HFD). Expression levels of *Tbx21, Gata3, Rorc, Foxp3,* and *Bcl6* in splenocytes from *Traf5*$^{+/+}$ (WT) and *Traf5*$^{-/-}$ (KO) mice fed either an HFD (**A**; see Figures 1B and 2A) or a normal diet (ND) (**B**; see Figures 1C and 2B) were quantified by real-time RT-PCR. Data are normalized to *Stx5a* expression and presented as mean ± standard error of the mean: (A) WT (n = 3), KO (n = 3); (B) WT (n = 5), KO (n = 5). For each gene, the expression level in WT mice was set to 1. *P*-values were calculated using Student's *t*-test.
